# The influence of exercise on body esteem among college students of different genders: evidence from Latin dance and aerobics

**DOI:** 10.3389/fpsyg.2025.1648594

**Published:** 2025-09-09

**Authors:** Yuna Hu, Yong Zeng, Heling Dong, Han Li

**Affiliations:** ^1^School of Physical Education, Jinan University, Guangzhou, China; ^2^Guangdong Provincial Key Laboratory of Speed Capability Research, Su Bingtian Center for Speed Research and Training, Guangzhou, China; ^3^College of Physical Education, Anhui Normal University, Wuhu, China

**Keywords:** body esteem, body image, physical self-perception profile (PSPP), Latin dance, aerobics, college students, gender differences, randomized controlled trial

## Abstract

**Background:**

Body esteem is pivotal to college students’ psychological well-being. Evidence suggests Latin dance and aerobics can enhance body image, but comparative, gender-specific effects remain underexplored.

**Methods:**

In a 12-week randomized controlled trial, students (*N* = 90; 45 men, 45 women) were assigned to Latin dance group (LDG), aerobics group (AG), or control group (CG). Body esteem was assessed pre- and post-intervention using PSPP subscales: sport competence (SC), physical condition (PC), body attractiveness (BA), physical strength (PS), and general physical self-worth (PSW). Statistical analyses included independent sample t-tests, paired sample t-tests, and ANOVA.

**Results:**

Both LDG and AG improved overall body esteem versus CG (*p* < 0.05). Compared with CG, LDG produced greater gains in BA and SC (*p* < 0.05), with significant benefits among female participant (BA, SC: *p* < 0.05). AG produced larger improvements in PC and PS versus CT (*p* < 0.05), with significant gains among male participant (PS: *p* < 0.01; PC: *p* < 0.05). No between-group differences were observed for PSW (LDG vs. AG, ns).

**Conclusion:**

This study indicates that both Latin dance and aerobics effectively enhance body esteem among college students but through distinct mechanisms. Latin dance particularly improves emotional and social dimensions of body esteem, offering pronounced benefits for female participant, while aerobics primarily enhances physical fitness aspects, more effectively benefiting male participant. These findings underscore the importance of providing diverse exercise modalities in university wellness programs to cater to varied gender-specific psychological and physical health needs.

**Clinical trial registration:**

ChiCTR25063201.

## 1 Introduction

Body esteem, which refers to the self-evaluation of one’s appearance and body shape, is crucial to an individual’s psychological health and overall well-being, especially for college students (Merino et al., 2024; Pop and Ciomag, 2024; Wang et al., 2025). During this transitional period in life, college students are often subject to social pressure and unrealistic aesthetic standards (Biderman et al., 2025; Merino et al., 2024), which may lead them to develop a negative body image and self-esteem (Bahri, 2024). Existing studies have demonstrated that physical activity is an effective intervention measure to enhance body esteem and has shown positive effects (Hale et al., 2021; Herbert, 2022; Morales-Sanchez et al., 2021; Singh et al., 2023). Among them, Latin dance and aerobic exercise are popular due to their engaging nature and potential health benefits (Hu et al., 2024; Li et al., 2023; Liu and Zhao, 2022; Liu and Zhao, 2022; Shu et al., 2023). However, there is a gap in comparative research on the specific effects of Latin dance and aerobic exercise on college students’ body esteem.

### 1.1 The impact of Latin dance on body esteem

Latin dance programs are often performed in pairs or groups and are social in nature, creating a sense of community and belonging (Harman, 2018). They are forms of movement that incorporate movement, music, and cultural expression, including rumba, cha-cha, samba, paso doble, and jive. These dances are often characterized by dynamic, fluid movements that emphasize rhythm, coordination, and self-expression (Li et al., 2023; Li et al., 2022). This social element can also positively promote interaction, boost confidence and improve body esteem (Sanchez, 2020). Dance, especially Latin dance, has been shown to have a positive impact on mental health and body image (Koch et al., 2014; Liu et al., 2023; Monteiro et al., 2018; Moratelli et al., 2023). Latin dance emphasizes body control (Li et al., 2023), posture (Li et al., 2022), and grace (Hu et al., 2024), which may help individuals develop a positive relationship with their bodies. Additionally, the social interactions inherent in Latin dance classes can reduce feelings of isolation and increase feelings of acceptance and self-worth (Ingram, 2013; Salo, 2019). People who participate in dance have higher levels of body satisfaction and self-esteem than those who engage in other forms of exercise. Additionally, Latin dance promotes creativity (Li et al., 2023) and self-expression (Ege and Omuris, 2024), which are important aspects of mental health. Moving freely, expressing oneself through dance (Shapiro, 2008), and embracing one’s cultural heritage may create a sense of empowerment that can improve body esteem. Research has also highlighted the benefits of Latin dance in fostering a positive body image, as it encourages people to embrace their bodies’ abilities rather than focusing solely on their appearance. This shift in focus may help improve body esteem by promoting a more holistic view of the body.

### 1.2 The impact of aerobics on body esteem

Aerobics is a form of high-intensity exercise with structured movements that focuses on cardiovascular fitness, endurance, strength and flexibility (Wilmore, 2003). Aerobics helps improve physical fitness (Chekhovskaya, 2023), improved mood (Liu et al., 2024), and reduced anxiety (Aishwarya and Kumar, 2024). Aerobics has also been associated with improved self-esteem and improved body image (Kuang, 2024), as individuals experience tangible improvements in their fitness levels and appearance. Aerobics can help control weight, muscle tone, and overall physical health (Riaz et al., 2024), which can have a positive impact on how individuals view their bodies. Additionally, the structured movement nature of aerobics involves setting goals, tracking progress, and achieving milestones, which can create a sense of accomplishment and boost self-esteem (Kuang, 2024). One study found that aerobics can significantly reduce body dissatisfaction, especially in those who previously had low body esteem (Henry et al., 2006).

However, compared to dance, aerobics may lack the same level of emotional and social engagement. While aerobics can indeed improve body image through physical changes, the psychological benefits related to self-expression, social connection, and cultural identity may not be as prominent. For many participants, the focus of aerobics may be primarily on fitness goals rather than the enjoyment of exercise or self-expression. So while aerobics does improve body esteem, the magnitude of this improvement may not be the same as the benefits of more expressive forms of exercise, such as Latin dance.

### 1.3 Gender differences in body esteem and physical activity

Gender differences in body esteem are well established, with female participant often reporting lower body esteem than male participant (Hagger and Stevenson, 2010; Kling et al., 1999), especially in cultures where appearance is highly valued. In contrast, male participant may be more concerned with muscularity and physical strength, but these concerns are often less pronounced than the body image pressures female participant face. The effects of physical activity on body esteem may differ by gender due to differences in psychological experiences and social expectations (Ómarsson, 2013). Female participant tend to gain greater body esteem through outward-focused activities (dance or aerobics) (Wang et al., 2025), male participant, on the other hand, may focus more on the functional aspects of exercise, such as strength and endurance. Latin dance, with its emphasis on fluidity, posture, body awareness, and partnering, may offer a more holistic way for female participant to improve their body esteem because it fosters self-expression and appreciation of the body, rather than just physical fitness. In contrast, aerobics may be seen as more practical and improve physical condition, but may lack the same level of emotional investment for women, which may result in less noticeable changes in their body esteem. For male participant, aerobics classes often focus on athletic performance goals such as endurance or weight loss, which can resonate with men’s body image issues, especially those related to a lean, toned physique. On the other hand, most men are not very supportive of Latin dance because of cultural perceptions that dance is a more feminine activity, which may limit its impact on the body esteem of male participant participants. However, over time, these perceptions may fade as dance is adopted as a unisex form of fitness.

Therefore, the main purpose of this study was to compare the effects of 12 weeks of Latin dance and aerobics training on body esteem among college students of different genders. Given that Latin dance and aerobics are both effective forms of exercise, understanding how they affect body esteem differently in male participant and female participant can provide recommendations for promoting psychological and emotional health in college students. This randomized controlled trial will provide empirical evidence on the psychological benefits of these two forms of exercise, with a particular focus on their effects on body esteem in different gender groups. Our hypotheses are as follows:

*H1*: Both Latin dance and aerobics will lead to significant improvements in body esteem compared to baseline measurements.

*H2*: The improvement in body esteem in the Latin dance group will be greater than that in the aerobics group.

*H3*: There are gender differences in the improvement in body esteem, with female participant achieving greater improvements through Latin dance and male participant achieving greater improvements through aerobics.

## 2 Materials and methods

### 2.1 Participants

**Figure 1:**
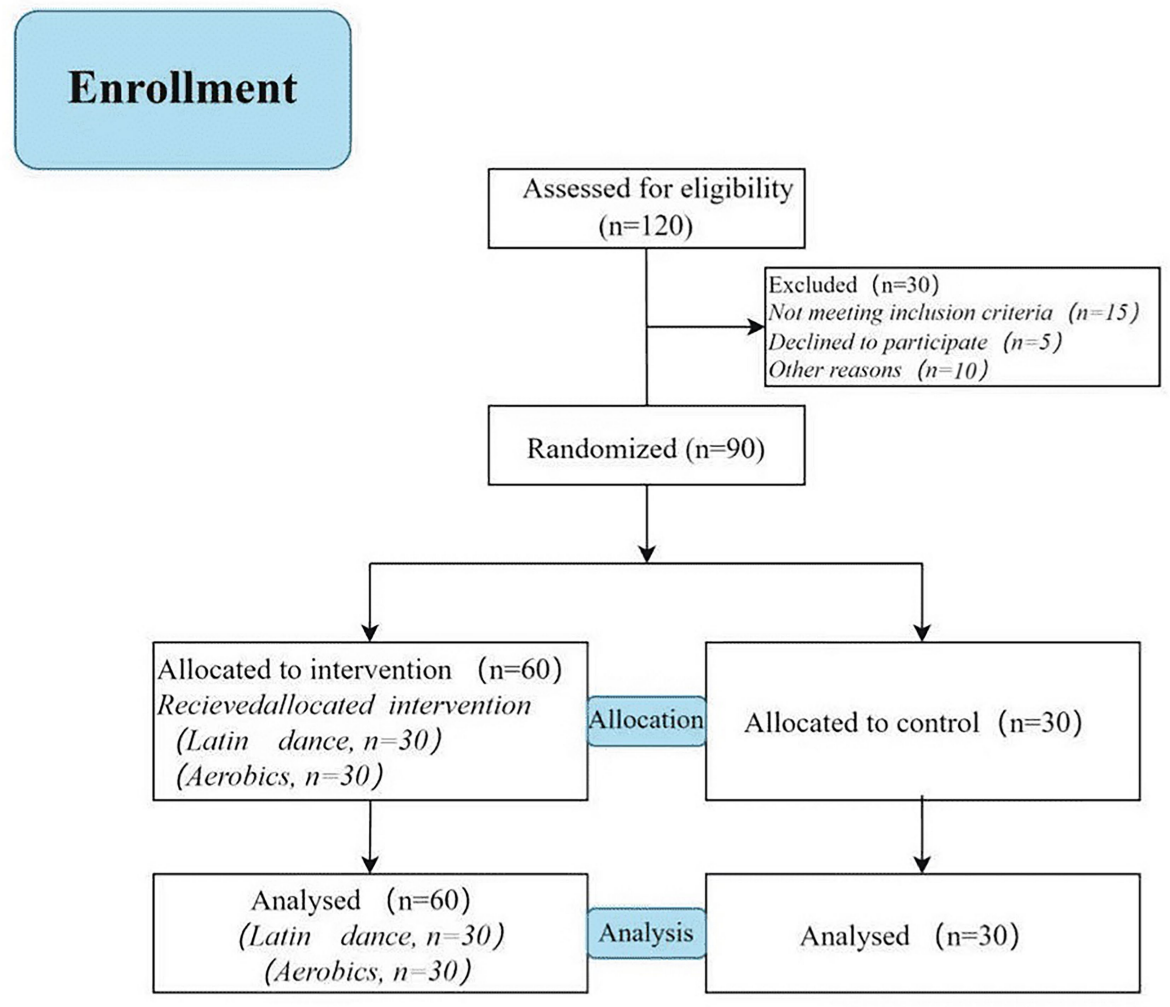
Study flowchart.

This study was conducted in the Latin dance and aerobics club of Yichun University, China. Prior to recruiting participants, an *a priori* efficacy analysis was conducted using G*Power 3.1 to determine the minimum sample size required for this study. The following parameters were set: effect size *f* = 0.23 (between small and medium), significance level *α* = 0.05, expected efficacy 1 - *β* = 0.85, repeated measures correlation coefficient *r* = 0.50, and non-spherical correction factor *ε* = 1. Calculations indicated that a total sample size of at least *N* = 84 would be needed. We selected 90 first-year university students who voluntarily participated in the club, including 45 male participants and 45 female participants (Aged between 18 and 21). The 45 male participant students were randomly divided into three groups: 15 Latin dance training for male participants (LTM), 15 aerobics training for male participants (ATM), and 15 control groups (Control for male participants, CM); the 45 female participants were randomly divided into three groups: 15 Latin dance training for female participants (LTF), 15 aerobics training for female participants (ATF), and 15 control groups (Control for female participants, CF). Inclusion criteria: no serious cardiovascular, respiratory, musculoskeletal diseases or other chronic diseases that may affect participation in sports training; after basic physical examinations or health questionnaires, it was confirmed that the basic physical conditions for participating in sports were met. Exclusion criteria: patients with severe mental disorders (such as major depression, anxiety, etc.); patients who are currently receiving relevant medications; patients with eating disorders and other behavioral problems that affect body image and self-esteem. Participants in each group underwent Functional Movement Screen (FMS) tests (Cook et al., 2014) before training, and no differences were found between the groups (Table 1). Figure 1 shows the progress of enrollment. All participants signed informed consent forms approved by the Hunan Normal University Ethics Committee (Approval ID: 2024646).

**Table 1:** Basic information of training participants.

Index	Gender	LDG	AG	CG	*F*	*p*
Age	Male	18.53±0.41	18.61±0.37	18.71±0.41	0.654	>0.05
	Female	18.97±0.47	18.64±0.34	18.77±0.42	0.614	>0.05
FMS test (score)	Male	15.35±2.48	15.17±2.27	15.34±2.18	0.248	>0.05
	Female	14.81±1.72	14.64±1.88	15.02±1.62	0.694	>0.05
Body esteem (score)	Male	71.46±9.17	72.14±10.68	71.80±7.82	1.679	>0.05
	Female	69.06±8.64	70.16±9.43	67.52±7.46	1.164	>0.05

### 2.2 Intervention

The experiment lasted for 12 weeks. The courses of exercise intervention were Latin dance and aerobics. Participants attended three classes per week, each class lasted 2 hours, with a 10-min break in between. The content of Latin dance training courses included basic steps, single and double combination routines, and dance music understanding exercises. The content of aerobics courses included basic steps, complete movement routines, and creation exercises. Each course was divided into three stages: basic (weeks 1–2), consolidation (weeks 3–7), and improvement (weeks 8–12). During the intervention, as the teaching content progressed, the intensity of the teaching class gradually increased. Since the teaching time was the same, the intensity of Latin dance and aerobics teaching was appropriately controlled to ensure that the difference in exercise load intensity in the two teaching classes was not large. During the training period, as the difficulty of the teaching content increased, the load intensity gradually increased. The specific intensity control is shown in Tables 2, 3. In order to evaluate the teaching effect and quality of the teachers, the participants presented their learning results after the entire training.

**Table 2:** Males’ exercise load control (*N* = 30).

Group	LTM	ATM	*T*	*p*
HR (1–4 week)	107.48 ± 12.79	110.83 ± 13.45	-1.437	>0.05
HR (5–8 week)	126.78 ± 11.79	121.81 ± 12.91	1.672	>0.05
HR (9–12 week)	144.42 ± 16.79	142.94 ± 16.43	1.261	>0.05

**Table 3:** Females’ exercise load control (*N* = 30).

Group	LTF	ATF	*T*	*p*
HR (1–4 week)	110.42 ± 14.64	116.14 ± 14.92	−1.67397	>0.05
HR (5–8 week)	134.93 ± 15.97	132.81 ± 15.66	1.297	>0.05
HR (9–12 week)	148.34 ± 16.72	147.23 ± 16.27	1.087	>0.05

### 2.3 Measurements

The Physical Self-Perception Profile (PSPP), developed by Kenneth R. Fox in 1990, is a multidimensional psychometric instrument designed to measure an individual’s self-perceptions related to their physical characteristics and abilities (Fox, 1990). PSPP has been widely used in many studies (dos Bucco Santos et al., n.d.; Chair et al., 2024; Jati et al., 2024; Nicolosi et al., 2024). It comprises five subscales—Sport Competence (SC), Physical Condition (PC), Body Attractiveness (BA), Physical Strength (PS), and General Physical Self-Worth (PSW)—each with six forced-choice items (30 items total). For each item, respondents choose between two contrasting statements and then indicate whether the choice is “really true” or “sort of true”; items are scored 1–4, yielding subscale totals of 6–24, with higher scores reflecting more positive perceptions. Subscales may be analyzed separately or jointly to assess domain-specific and overall physical self-esteem and to evaluate the effects of physical activity or interventions on physical self-concept.

### 2.4 Statistical methods

All data were statistically analyzed using IBM SPSS Statistics 30.0.0 software. To evaluate intra-group changes and inter-group comparisons, independent sample t tests, paired sample t tests, and one-way analysis of variance (ANOVA) were used. Data were described as (mean ± standard deviation) and statistically significant accepted *p* < 0.05. For the differences between groups after ANOVA, *post hoc* tests were performed using the Bonferroni method and the corrected *p*-value was reported. The following are the statistical analysis methods used for each hypothesis:

*H1*: Use a paired t test to evaluate changes in body self-esteem before and after Latin dance and aerobic exercise.

*H2*: The differences in the Latin dance and aerobic exercise groups in terms of body attractiveness (BA) and motor ability (SC) were compared using independent sample t test or one-way analysis of variance (ANOVA).

*H3*: Use independent sample t test or gender-based variance analysis to compare the effects of different genders in two forms of exercise.

## 3 Results

### 3.1 Changes in body esteem before and after Latin dance training

After 12 weeks of Latin dance training, the changes in various indicators of body esteem are shown in Table 4.

**Table 4:** Results of indicators pre- and post-Latin dance intervention.

Variables	Groups	Pre-test	Post-test	*T*	*p*
PSPP	LDG	70.86 ± 11.65	83.95 ± 9.45	2.339	<0.05*
PSPP	LTM	71.46 ± 9.17	87.46 ± 8.27	2.681	<0.01**
	LTF	69.06 ± 8.64	80.68 ± 8.24	2.166	<0.05*
PSW	LDG	13.55 ± 3.45	15.94 ± 2.28	1.981	<0.05*
PSW	LTM	13.87 ± 2.79	16.87 ± 2.82	2.342	<0.05*
	LTF	13.13 ± 2.49	14.83 ± 2.61	1.762	>0.05
SC	LDG	11.43 ± 2.46	15.46 ± 2.46	2.342	<0.05*
SC	LTM	11.93 ± 3.80	16.03 ± 2.91	2.842	<0.01**
	LTF	11.74 ± 3.31	15.04 ± 2.41	2.143	<0.05*
PC	LDG	15.49 ± 2.87	16.56 ± 2.73	1.834	>0.05
PC	LTM	15.94 ± 2.28	18.54 ± 2.32	2.342	<0.05*
	LTF	15.51 ± 3.49	15.41 ± 2.53	1.513	>0.05
BA	LDG	14.33 ± 3.16	17.38 ± 2.34	2.438	<0.05*
AB	LTM	14.25 ± 3.65	17.25 ± 3.82	2.341	<0.05*
	LTF	15.44 ± 2.16	17.44 ± 2.36	2.168	<0.05*
PS	LDG	14.83 ± 2.73	18.66 ± 2.35	2.634	<0.01**
PF	LTM	14.89 ± 3.43	18.86 ± 3.13	2.661	<0.01**
	LTF	14.27 ± 3.46	18.27 ± 2.62	2.653	<0.01**

Data are presented as the mean ± sd, *p < 0.05, **p < 0.01.

**Figure 2:**
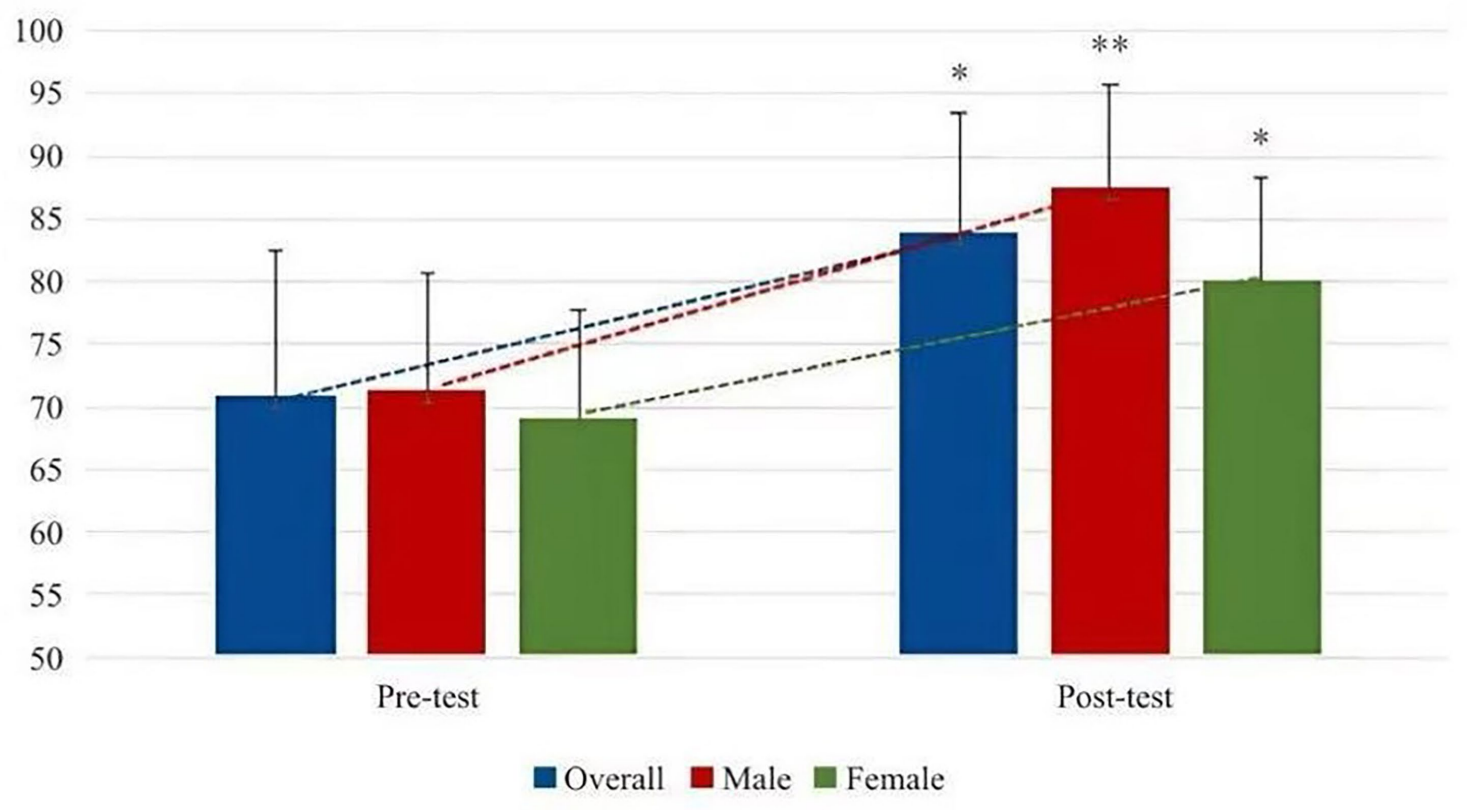
Changing trend of the effect of Latin dance training on body esteem.

**Figure 3:**
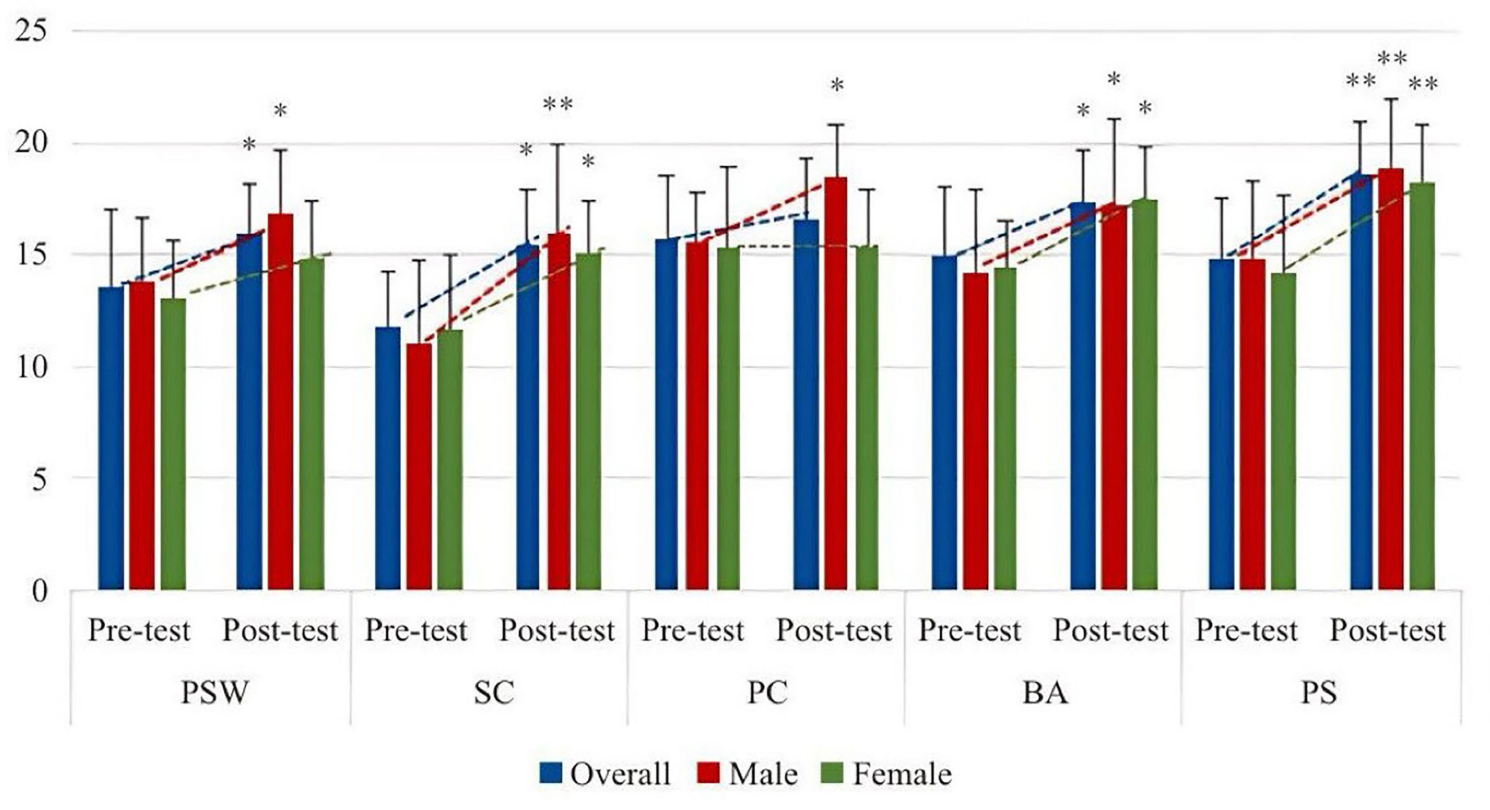
The changing trend of the effects of Latin dance training on various dimensions of body esteem.

From Table 4, Latin dance teaching significantly improves students’ body self-esteem, and male participants are ahead of female participants. In the five small dimensions, except for the evaluation of physical condition, the other dimensions are significantly improved. In the longitudinal comparison of PC, male participants have improved significantly, while female participants have not significantly changed and have slightly decreased. In the other four dimensions, male participants’ physical self-esteem and SC have improved faster than female participants, and the difference in female participants’ physical self-esteem is not significant. In terms of BA and PS, the improvement of male participants and female participants is basically the same, and the improvement of PS is very obvious. The specific improvement trend is shown in Figures 2, 3.

### 3.2 Difference analysis of body esteem after the experiment

#### 3.2.1 Comparison of specific scores of body esteem levels after the experiment

**Figure 4:**
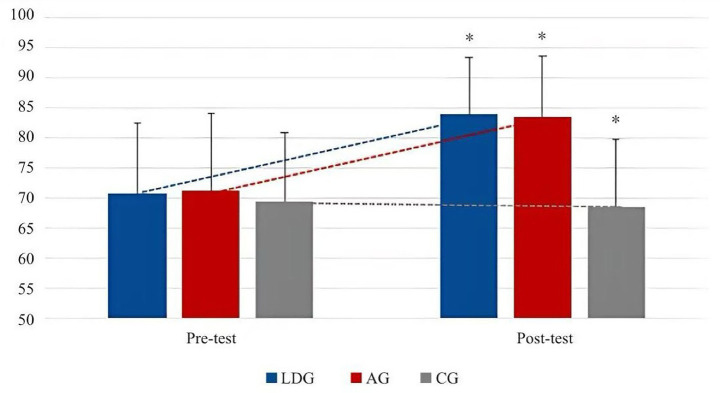
Change trend of total scores of body esteem between pre- and post-test.

**Figure 5:**
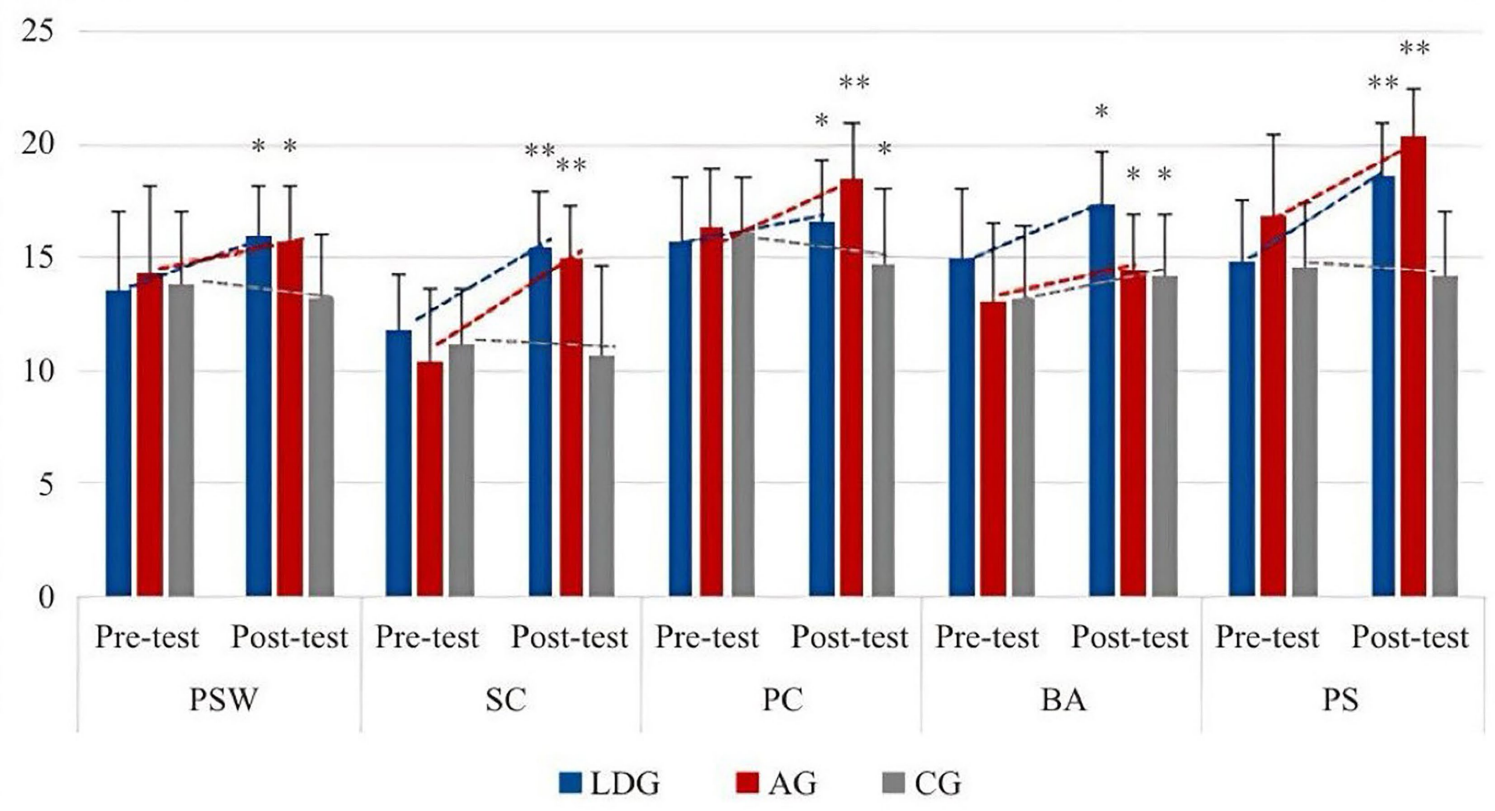
The trend of scores of each dimension of body esteem between pre- and post-test.

From Table 5, there was no significant difference between LDG and AG (original *p* > 0.05), but compared with CG, there was a significant difference (adjusted *p* < 0.05, corrected by Bonferroni). On each dimension, the difference in PSW was *p* > 0.05, with no significant difference, but the difference between the two groups and the control group was *p* < 0.05, and the difference was significant after Bonferroni correction, but the difference between the two groups and the control group is *p* < 0.05, which is significantly different from the control group; in the evaluation of PC, the difference *p* value between each two groups is less than 0.05, and the difference is significant. The order of evaluation level from high to low is AG, LDG, CG, in the evaluation of BA, the difference p value between each two groups is less than 0.05, and the difference is significant, and the order from high to low is LDG, AG, CG; in the evaluation of PS level, the difference between AG and CG is *p* < 0.01, and the difference is very significant. The *p* values of the other two groups are less than 0.05, and the difference is significant. The order of evaluation level from high to low is AG, LDG, CG. The changing trends of the total score and each dimension of body esteem in each group are shown in Figures 4, 5.

**Table 5:** Comparative analysis of body self-esteem levels among groups after the experiment (M ± SD).

Variables	Latin Dance group (LDG)	Aerobics group (AG)	Control group (CG)	*F*	*p*	*Bonferroni*
PSPP	83.95 ± 9.45	83.46 ± 10.27	69.46 ± 11.43	2.911	<0.05*	LDG > AG AG > CG* LDG > CG*
PSW	15.94 ± 2.28	15.47 ± 3.49	13.44 ± 2.84	2.603	<0.05*	LDG > AG AG > CG* LDG > CG*
SC	15.46 ± 2.46	15.03 ± 2.23	11.53 ± 3.89	2.661	<0.05*	LDG > AG AG > CG* LDG > CG*
PC	16.56 ± 2.73	18.36 ± 3.48	15.97 ± 3.31	2.713	<0.05*	LDG < AG* AG > CG* LDG > CG*
BA	17.38 ± 2.34	14.49 ± 3.38	13.28 ± 2.75	2.867	<0.05*	LDG > AG AG > CG* LDG > CG*
PS	18.66 ± 2.35	20.36 ± 2.18	14.74 ± 2.78	3.416	<0.01**	LDG < AG* AG > CG** LDG > CG*

Data are presented as the mean ± sd, *p < 0.05, **p < 0.01.

#### 3.2.2 Comparison of body esteem scores between different genders after the experiment

##### 3.2.2.1 Comparison of body esteem scores of male participant after the experiment

From Table 6, we can see that in the comparison of body self-esteem between LTM and ATM, the *p* value of the overall self-esteem level is greater than 0.05, which does not reach a statistically significant difference. In the five small dimensions, the *p* values of PSW, SC, PC, and BA are all greater than 0.05, which does not reach a statistically significant difference. Only PS shows a significant difference. Combined with the level before the experiment, LTM and ATM are ahead of CG in overall body self-esteem.

**Table 6:** Comparative analysis of the differences in body self-esteem among males in each group after the experiment (M ± SD).

Variables	LTM	ATM	CM	*F*	*p*	*Bonferroni*
PSPP	87.46 ± 8.27	90.14 ± 9.38	70.14 ± 8.54	3.719	<0.01**	LTM < ATM ATM > CM** LTM > CM*
PSW	16.87 ± 2.82	17.07 ± 2.64	14.14 ± 2.32	2.851	<0.05*	LTM < ATM ATM > CM* LTM > CM*
SC	16.03 ± 2.91	15.41 ± 2.26	11.34 ± 3.55	2.811	<0.05*	LTM > ATM ATM > CM** LTM > CM**
PC	18.54 ± 2.32	18.76 ± 2.22	16.27 ± 2.73	2.637	<0.05*	LTM < ATM ATM > CM* LTM > CM*
BA	17.25 ± 3.82	17.98 ± 2.26	13.42 ± 3.71	2.961	<0.05*	LTM < AM ATM > CM** LTM > CM**
PS	18.86 ± 3.13	20.89 ± 2.31	14.83 ± 3.72	3.711	<0.01**	LTM < ATM* ATM > CM** LTM > CM**

Data are presented as the mean ± sd, *p < 0.05, **p < 0.01.

**Figure 6:**
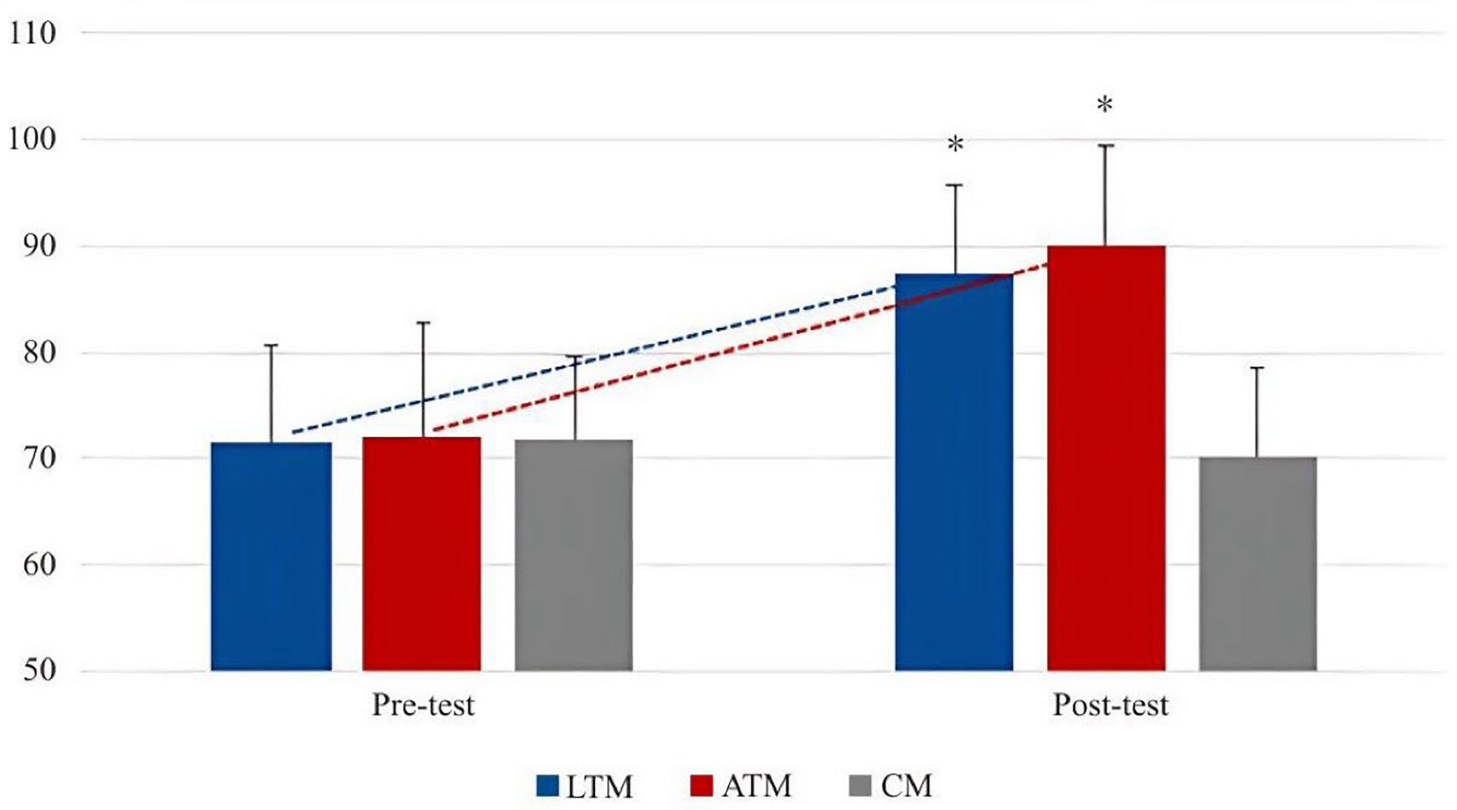
The trend of the total scores of body esteem in each group of males between pre- and post-test.

**Figure 7:**
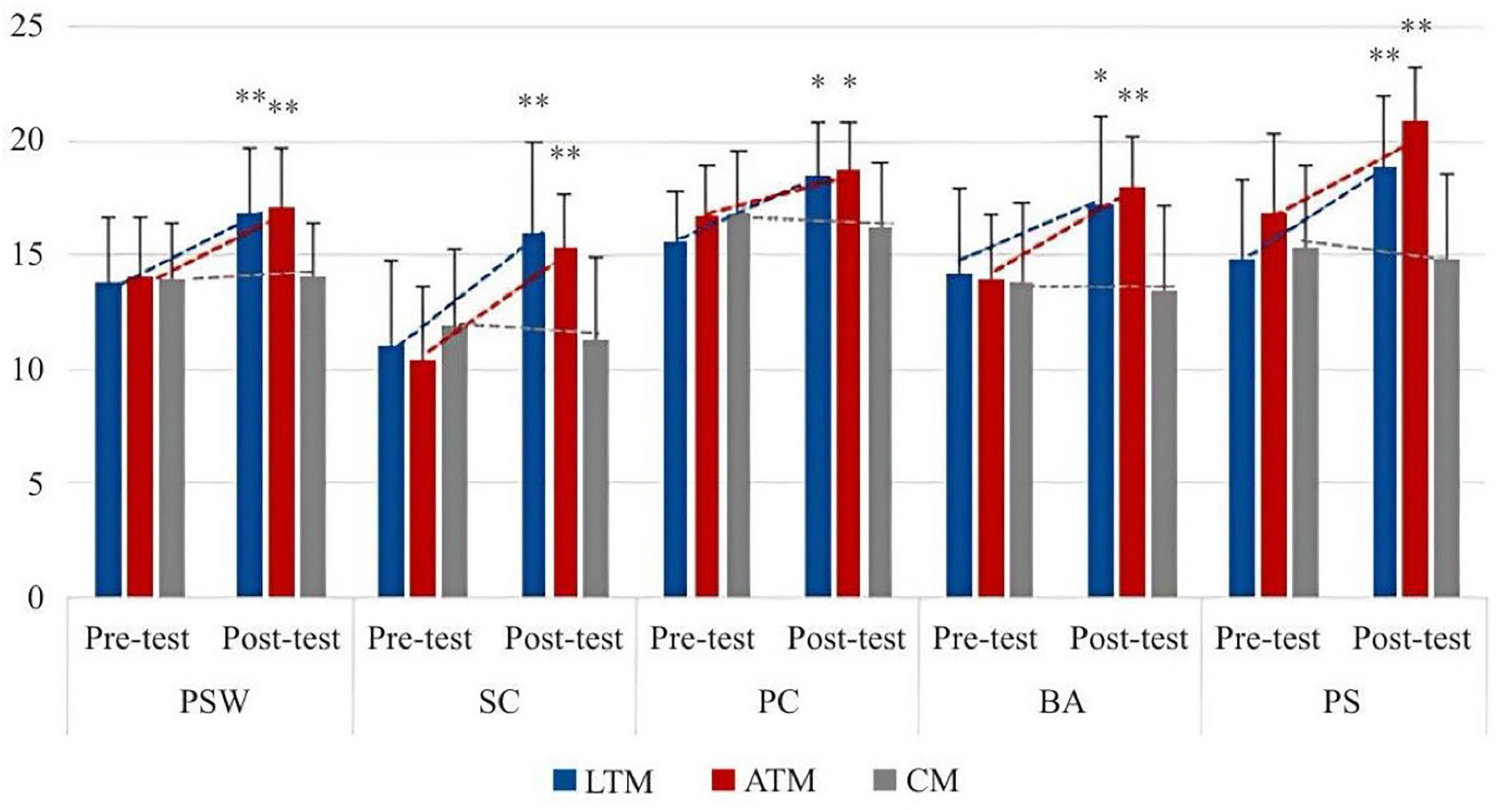
Change trend of body esteem scores in each group of males between pre- and post-test.

In general, the overall level of body esteem of ATM was slightly higher than that of LTM, but there was no significant difference. From a small dimension, Latin dance and aerobics both had a greater ability to improve SC, PC, and PS, and had a smaller improvement on PSW and BA. Latin dance was slightly better than aerobics in improving SC and PC, and aerobics was slightly better than sports dance in improving BA and PS. The changing trend is shown in Figures 6, 7.

##### 3.2.2.2 Comparison of body esteem scores of female participant after the experiment

From Table 7, after the experiment, in the evaluation of female participant’s body self-esteem level, LTF was slightly higher than ATF, which did not form a statistically significant difference (*p* > 0.05), but the difference was significant compared with CF (*p* < 0.05). In the comparison of small dimensions, in the evaluation of PSW, there was no statistical difference between ATF and LTF and CF (*p* > 0.05), but there was a significant difference between LTF and CF (*p* < 0.05). In the SC evaluation level, there was no significant difference between the LTF and ATF groups (*p* > 0.05), but the difference between the two groups and CF was *p* < 0.05, which was significantly better than the control group. In the PC evaluation level, ATF was significantly different from the other two groups (*p* < 0.05). In the BA, the difference in the evaluation level between each two groups was *p* < 0.05, and the difference was significant. The order from high to low was LTF, CF, and ATF. In the PS evaluation level, the average levels of ATF and LTF were high, among which the difference between ATF and CF was very significant (*p* < 0.01), the difference between LTF and CF was significant (*p* < 0.05), and there was no significant difference between LTF and ATF (*p* > 0.05).

**Table 7:** Comparative analysis of the differences in body self-esteem among females in each group after the experiment (M ± SD).

Variables	LTF	ATF	CF	*F*	*p*	*Bonferroni*
PSPP	80.68 ± 8.24	77.96 ± 9.43	68.42 ± 7.46	2.991	<0.05*	LTF > ATF ATF > CF* LTF > CF*
PSW	14.83 ± 2.61	14.39 ± 2.13	13.77 ± 2.72	2.636	>0.05	LTF > ATF ATF > CF LTF > CF*
SC	15.04 ± 2.41	14.01 ± 2.79	11.09 ± 3.38	2.901	<0.05*	LTF > ATF ATF > CF* LTF > CF*
PC	15.41 ± 2.53	18.18 ± 2.35	15.83 ± 2.36	2.684	<0.05*	LTF < ATF* ATF > CF* LTF < CF
BA	17.44 ± 2.36	11.27 ± 2.64	13.07 ± 3.35	3.279	<0.05*	LTF > ATF* ATF < CF* LTF > CF*
PS	18.27 ± 2.62	19.93 ± 2.78	14.69 ± 3.52	3.616	<0.01**	LTF < ATF* ATF > CF** LTF > CF*

Data are presented as the mean ± sd, *p < 0.05, **p < 0.01.

**Figure 8:**
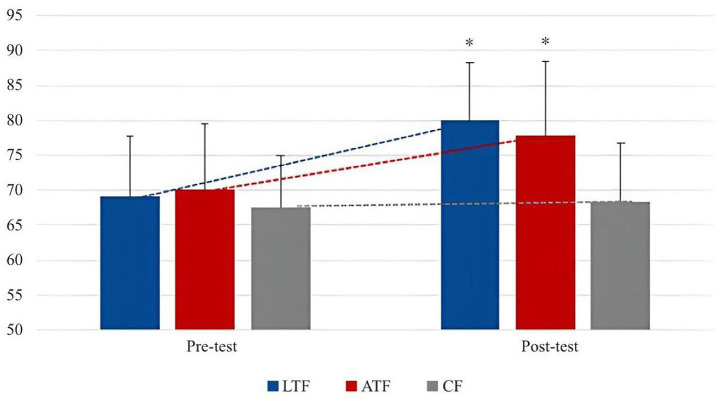
The trend of the total scores of body esteem in each group of females between pre- and post-test.

**Figure 9:**
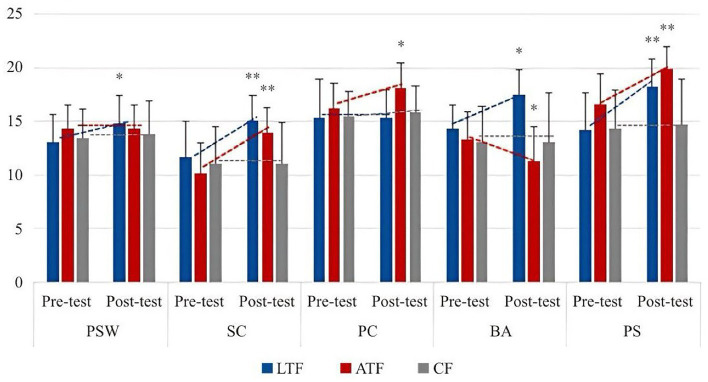
Change trend of body esteem scores in each group of females between pre- and post-test.

In general, after the experiment, the body self-esteem of LTF and ATF was significantly better than that of CF, but not as obvious as that of boys. In terms of small dimensions, ATF had a large gap with LTF in PC and PS, but LTF had a significant advantage in the evaluation of BA. In terms of PSW and SC, the difference between the two was not large, and it was better than the pre-test level in all dimensions. The changing trend is shown in Figures 8, 9.

## 4 Discussion

The present study aimed to evaluate the effects of Latin dance compared to aerobics on body esteem among college students and to explore potential gender differences in these effects. The results clearly demonstrate significant improvements in body esteem following both exercise interventions, which aligns with existing literature supporting physical activity as a beneficial intervention for body image and self-esteem enhancement (Fernandez-Bustos et al., 2019; Huang et al., 2007; Morales-Sanchez et al., 2021; Rusu et al., 2025; Teixeira et al., 2025). Importantly, the improvements varied between Latin dance and aerobics across several dimensions of body esteem, suggesting distinct underlying mechanisms and implications for practice.

### 4.1 Overall effects of Latin dance and aerobics

Our findings indicate substantial improvements in overall body esteem for participants engaged in Latin dance and aerobics compared to the control group. Both forms of exercise significantly elevated students’ general physical self-worth, sports competence, physical condition, body attractiveness, and physical strength, reinforcing previous findings that structured physical activities positively impact psychological health and body perceptions (Edwards et al., 2005; Fox, 2003; Gualdi-Russo et al., 2022). The pronounced improvement observed underscores the integral role of regular physical activity in fostering a positive self-image and enhancing mental well-being among college students, a population frequently vulnerable to body dissatisfaction and self-esteem issues (Pastore et al., 2015; Pop, 2016).

### 4.2 Comparative analysis of Latin dance versus aerobics

Although both the Latin dance and aerobic groups showed improvements, the Latin dance participants showed a more significant improvement in physical attractiveness and athletic ability (corrected *p*-value < 0.05, Bonferroni method). Aerobic exercise showed significant improvements in physical fitness and strength (corrected *p*-value < 0.05, Bonferroni method). A plausible explanation is that in Latin dance, rhythmic control and postural attention help them to focus on physical aesthetics and motor skills; these traits may reinforce positive evaluations of appearance and perception (Hu et al., 2024; Li et al., 2023). This outcome resonates with previous research highlighting dance’s unique role in enhancing body image through creative and social engagement (Bicenturk, 2024; Grogan et al., 2014).

Conversely, aerobics training yielded greater gains in physical condition and physical strength dimensions, reflecting its structured, fitness-oriented nature that emphasizes endurance, muscular strength, and cardiovascular health (Schjerve et al., 2008). The goal-oriented and progressively challenging aspects of aerobics may contribute significantly to these particular improvements, aligning with studies suggesting aerobic activities effectively boost physical health perceptions and body functionality (Wilmore, 2003).

### 4.3 Gender-specific effects

An essential finding from our study is the observed gender differences in response to the exercise interventions. Male participants demonstrated greater improvements in overall body esteem, particularly in physical strength and physical condition dimensions, after aerobics training compared to Latin dance. Compared to Latin dance, aerobic exercises may better align with most men’s focus on fitness and performance due to their functional, progressive, and muscular endurance characteristics (Hands et al., 2016). However, given that the evidence is limited to the subdimensions of PS and PC, this explanation still requires caution and cannot directly conclude that men generally prefer functionality.

Female participants, on the other hand, exhibited pronounced improvements in body attractiveness and sports competence from Latin dance training. Latin dance likely addresses the societal pressures women frequently encounter regarding appearance, offering a holistic approach that incorporates emotional (Quiroga Murcia et al., 2010) and social elements (Green, 2001) along with physical fitness. These findings corroborate previous research that suggests expressive physical activities like dance have significant psychosocial benefits for women, promoting acceptance, self-expression, and enhanced body image (Crosby, 2013; Fourie and Lessing, 2010; Pandya, 2024).

### 4.4 Mechanisms and practical implications

The observed differential benefits can be attributed to inherent characteristics of each exercise modality. Latin dance, with its emphasis on rhythm, coordination, and social interaction, fosters emotional connection, community belonging, and expressive freedom (Liu et al., 2023), which might indirectly elevate body esteem by reducing psychological stress and promoting positive social interactions (Li, 2023).

Aerobics, with its structured progression and tangible fitness outcomes, enhances self-efficacy, physical health perceptions, and accomplishment. The clear progression and achievement of fitness goals in aerobics provide measurable feedback that directly boosts physical self-worth and strength perceptions, particularly resonating with those valuing clear fitness outcomes (Kim et al., 2025).

From a practical standpoint, educators and fitness professionals should consider integrating both Latin dance and aerobics into physical education curricula and campus wellness programs to cater to diverse student needs and preferences. Specifically, Latin dance could be recommended for individuals seeking psychosocial benefits and improved body attractiveness, while aerobics may be more suited for those prioritizing fitness goals and physical strength.

### 4.5 Limitations and future directions

Despite its contributions, this study has certain limitations. The relatively small sample size and short intervention period might have constrained the comprehensiveness of the outcomes. Additionally, cultural perceptions influencing the gendered response to exercise modalities were not explicitly measured and could be explored further. Future research should consider longitudinal designs to evaluate sustained effects, larger diverse populations for greater generalizability, and qualitative analyses to understand deeper psychosocial impacts.

## 5 Conclusion

In conclusion, this study demonstrates significant benefits of both Latin dance and aerobics on body esteem in college students, highlighting distinct gender-specific responses to these exercise modalities. Latin dance uniquely enhances attractiveness and athletic ability, particularly benefiting female participants, whereas aerobics effectively boosts physical strength and conditioning, appealing especially to male participants. These findings underscore the importance of providing varied exercise options within college wellness programs to holistically support students’ psychological and physical health.

## Data Availability

The raw data supporting the conclusions of this article will be made available by the authors, without undue reservation.
